# Current Discussions on Employees and Organizations During the COVID-19 Pandemic: A Systematic Literature Review

**DOI:** 10.3389/fpsyg.2022.848778

**Published:** 2022-04-12

**Authors:** Soyeon Mun, Yoosun Moon, Hayeseul Kim, Namhee Kim

**Affiliations:** Department of Education, Ewha Womans University, Seoul, South Korea

**Keywords:** systematic literature review, COVID-19, human resources, employee wellbeing, organizational strategies

## Abstract

New concerns have emerged during the COVID-19 pandemic that greatly impact employees and many other aspects in organizations. We have highlighted the major organizational issues during COVID-19 and classified the relevant research findings based on 45 recent articles. Main themes identified include (1) work setting, (2) perceptions of COVID-19, (3) employee wellbeing, (4) organizational strategies, and (5) influences on career behaviors. Employees have faced challenges due to work conditions that have shifted from traditional commuting to telework. Employees have also become aware of the negative current situation, so their overall wellbeing has been threatened. In response, organizations have strived to promote positive psychological capital for employees as they attempt to cope with this crisis. Organizations have tried to maintain and manage both their employees and their business. People tended to adjust their career-related behaviors based on how they perceived their own wellbeing and organizational strategies.

## Introduction

The ongoing COVID-19 pandemic has had destructive effects on every aspect of people’s lives. Since the World Health Organization (WHO) declared COVID-19 an international pandemic, countermeasures responding to the crisis (e.g., the compulsory use of personal protective equipment, social distancing, and lockdown policies) have been implemented worldwide (World Health Organization [WHO], 2020). Accordingly, recent literature has paid attention to the concerns that have emerged in the wake of the COVID-19 pandemic in the workplace.

Given that workers have been challenged by new work conditions and changing lifestyles during COVID-19, scholars have begun to discuss various factors influencing employees’ job performance and changes caused by the COVID-19. For example, how employees perceive the COVID-19 situation has been considered an important variable affecting their job behavior and wellbeing (Kuang et al., 2020; Yan et al., 2021). When people face danger, their anxiety or fear of exposure to risk impacts their behavior (Bae and Chang, 2021). In addition, COVID-related predictors of employee distress have been explored, including loneliness, technostress, and work-home conflict in situations where they lack contact with others (Molino et al., 2020; Tuzovic and Kabadayi, 2021).

In terms of organizational sustainability, employers have faced novel problems related to organizational management and human resources since the pandemic struck. Many organizations have had to cut employees’ salaries and lay workers off to survive financially (Jasmine, 2020). Critical organizational concerns surrounding employee support have also been compounded by new guidelines for workers, for example, as many work experiences have changed (Chen and Eyoun, 2021). In addition, there has been growing interest in efficient telework systems, as employees and organizations have had to adjust to virtual and mobile work systems (Raišienė et al., 2020; Bartsch et al., 2021). To address these challenges, many studies have offered suggestions to provide support for employees and their organizations (Aguinis and Burgi-Tian, 2021; Kuknor and Bhattacharya, 2021).

Unpredictable changes triggered by the pandemic have been a popular subject in the organizational psychology since the virus emerged, resulting in numerous studies examining the ongoing challenges and changes from different perspectives. Considering the proliferation of relevant studies related to COVID-19 in the past 2 years, it is time to review and detect the overall trends and focus of these studies. In particular, it is important to summarize how employees suffered from psychological and behavioral challenges and how organizations addressed various problems. Examining these studies will uncover lessons learned and provide direction for future studies. Therefore, the purpose of this study is to synthesize the main topics on employees and organizations discussed in recent academic studies related to the COVID-19 pandemic using a systematic literature review. The findings can provide insights to help prepare for future challenges and highlight needed interventions at the employee and organizational levels.

## Methodology Design

### Literature Search

**FIGURE 1:**
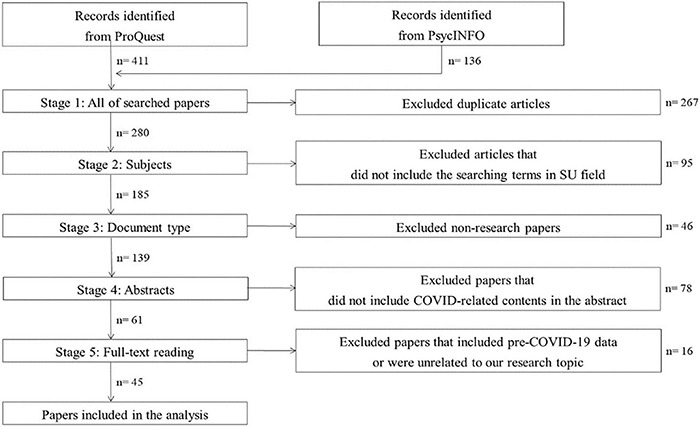
Research protocol.

A systematic literature review is to identify, evaluate, and interpret available research related to a specific area of interest (Fink, 2005). We adopted the overall procedures of a systematic literature review because it classifies previous themes from the literature. Following the PRISMA 2020 statement (Page et al., 2021), we specified the search protocol to select the literature (Figure 1). We selected ProQuest and PsycINFO as our search databases since they are common databases when researching in social sciences including psychology, business, and management. We searched articles in these databases from February to April 2021, using a combination of the subjects *human resources or employee* AND *covid or coronavirus*.

### Inclusion and Exclusion Criteria

Articles were included if they met the following criteria. We collected 280 articles using the standards in the search field of All subjects & indexing (SU) and full-text, peer-reviewed scholarly journals written in English after 2020, which corresponds to the pandemic period. Although these articles were searched based on the search criteria, we excluded 95 articles in the second stage because the search terms were not included in our target field. For example, Cotterill et al. (2020) was initially browsed when searched with our search criteria. However, although the term *employee* was included in other fields, it was not in the subject field. Next, we filtered 46 non-research articles such as editorials and commentaries in the third stage. Following the fourth stage, we removed 78 papers whose abstracts did not contain any discussion related to COVID-19. For example, the abstract of Bhandari et al. (2020) mentioned only about health workers’ core competencies that were not directly connected with COVID-19, so the article was excluded. Finally, we read the full text of each article and excluded 16 articles that used data prior to COVID-19 and were not specifically related to our topic. For example, Palumbo’s (2020) study was excluded because the data were collected in 2015. As a result of this process, 45 articles remained for the review based on the collected data.

The first three authors conducted the first search together following the search protocol described above. In the second stage, we divided the collected articles from stage 1 into three groups, and each author conducted the following screening process by checking the search terms in our target field. Throughout stages 3–5, to avoid bias, one author read the articles and the others cross-checked them to determine whether each article matched the search protocol and was relevant to the purpose of our study. After finishing this procedure, all authors discussed and reached consensus on whether each article should be included or excluded based on if the article discussed how employees and organizations responded to the pandemic.

Among the 45 articles, 34 related to employees and businesses in industry, 3 articles related to education, and 8 articles were from the medical field. The articles were not restricted to one country or region. As for the types of research, 7 studies were qualitative, 32 were quantitative, 2 used both qualitative and quantitative methods, and 3 were conceptual studies. In addition, a few articles synthesized problems related to both employees and organizations during COVID-19, although most papers provided a broad, general discussion of both.

### Research Theme Classification

Similar to the study selection process, the authors read the sample articles, identifying the main idea and summarizing them into one or two phrases. Then sub-themes were confirmed by organizing the common content that had been cross-checked. These sub-themes were grouped into superordinate concepts based on the common topics or focused variables. Final consensus was reached on five main themes: (1) Work setting; (2) Perceptions of COVID-19; (3) Employee wellbeing; (4) Organizational strategies; and (5) Influences on career behaviors (Table 1).

**TABLE 1:** Theme classifications.

Main theme	Sub-themes	Number of articles
1. Work setting	(1) Acceleration of work-setting transformation	2
	(2) Transition to new work contexts; work-from-home, returning to work	9
2. Perceptions of COVID-19	Impact of perceived COVID-19 anxiety on work performance	9
3. Employee wellbeing	(1) Employees’ adjustment	1
	(2) Employees’ mental health; [non-frontline] mental health problems, [frontline] mental health problems with vulnerable work conditions	8
	(3) Social support	2
4. Organizational strategies	(1) Leadership strategies	2
	(2) Organizational HR strategies	3
	(3) Organizational management strategies	5
	(4) Employees’ perceptions of organizational strategies	1
5. Influences on career behaviors	(1) The decision to be absent (absenteeism)	1
	(2) Future career decisions	2

## Results and Discussion

### Work Setting

Work setting entails the elements required for work to be done, such as goals, activities, tasks, and individual roles (Evdokiou and Vernik, 2011). This theme includes both job environments and work conditions (Table 2). Since telework was generally required for employees during the pandemic, adaptation was a new goal for organizations due to the acceleration of work-setting transformation and the transition to a new work environment. Therefore, we categorized the literature that examined changing work environments and difficulties transitioning to new work conditions after COVID-19.

**TABLE 2:** Articles on work setting.

Sub-themes	References	Research objective
(1) Acceleration of work-setting transformation	Abulibdeh (2020)	To examine the effects of COVID-19 mitigation actions on promoting telework implementation
	Nagel (2020)	To assess the acceleration of a digital working environment due to the pandemic
(2) Transition to new work circumstances, work-from-home, and returning to work	Bhattacharjee (2020)	To evaluate the applicability of working from home as an alternative to daily commuting for women in India
	Bhattacharya and Mittal (2020)	To analyze how the dominant desire of individuals impacts their WTHC or HTWC during telework
	Bolisani et al. (2020)	To identify employees’ experiences of working from home during COVID-19
	Diab-Bahman and Al-Enzi (2020)	To examine insights on the impact of working from home as a new condition
	Sethi and Saini (2020)	To investigate how schoolteachers experience working from home
	Song et al. (2020)	To assess mental health and work attitudes of employees who returned to work after the Spring Festival in China
	Tan et al. (2020)	To identify the mental health of workers and the effect of psychoneuroimmunity prevention when people returned to work during the pandemic
	Vaziri et al. (2020)	To explore employees’ profiles and changes in the work-home interface
	Yuan et al. (2021)	To investigate what factors promoted employees’ return to work and how leaders maintained engagement-enhancing effects

The work environment has changed since the beginning of the pandemic. Although some organizations promoted telework in the past, COVID-19 accelerated remote work for most employees. Nagel (2020) investigated whether COVID-19 has accelerated a digital transformation, and the number of people working from home has increased. Abulibdeh (2020) conducted a review of the impact of COVID-19 and revealed that telework has become an essential attribute of future organizations because telework practices have increased. People reported digital work will become more common, and want to telework more often (Abulibdeh, 2020; Nagel, 2020).

Employees’ experiences of working from home in various sectors during lockdowns and social distancing were examined in several studies as an alternative way to maintain business. Bolisani et al. (2020) explored both the positive and negative aspects of working from home and found that the response to working from home has two sides. They indicated that working from home is not always positive and can be inefficient when employees are forced to do so (Bolisani et al., 2020). According to Sethi and Saini (2020), schoolteachers who worked from home perceived their jobs positively even though they faced moderate challenges. In another study, flexible working hours were perceived as a benefit of working from home and an increased focus on work was also reported (Diab-Bahman and Al-Enzi, 2020). In contrast, in a study on women who worked from home in India (Bhattacharjee, 2020), the participants reported that working from home had some disadvantages, such as difficulty transitioning between their domestic role and working role, and the added burden of chores.

The sudden transition to working from home seemed to have caused more work-home conflict and confusion for many employees. For example, Vaziri et al. (2020) examined which people experienced more conflict when working from home. People who previously had low conflict and low enrichment from the work-family interface prior to COVID-19 experienced greater conflict after the COVID-19 outbreak while people with low prior conflict and high enrichment remained the same (Vaziri et al., 2020). Bhattacharya and Mittal (2020) found that employees who wanted to complete their work on time or preferred to sustain social connections experienced more work-home conflict.

Some scholars also focused on employees who returned to work after quarantine or lockdown. Tan et al. (2020) explored the psychological states of workers returning to work with the fear of contagion and the effect of psychoneuroimmunology prevention measures. The possibility of experiencing psychiatric symptoms was lower if employees sensed that there was improved workplace hygiene and that the organization was concerned about their health. Yuan et al. (2021) revealed the effect of job attachment as another factor that influenced the job engagement of employees who went back to work after lockdown. In addition, the effectiveness of leaders’ commitment to safety moderated the relationship between job attachment and engagement. Leaders’ commitment to safety also strengthened the effect of job attachment so employees were prepared to focus on work (Yuan et al., 2021). Similarly, Song et al. (2020) conducted an analysis based on risk factors and protective factors that affected workers’ mental health. Concern about unemployment was another risk factor that led to anxiety, depression, and insomnia while resilience and optimism were protective factors for mental problems. Thus, the researchers in the studies we reviewed argued that proper organizational intervention and attention are needed to reduce psychological difficulties for these employees.

The results show that more workers have been working from home, and that employees expect digital work environments to become a common and essential aspect of the future. Before the pandemic, telework was considered a favored alternative to the traditional setting of working in an office and was perceived as progress in business management and technology (Greer and Payne, 2014). In this process of development, telework has become a fundamental part of work as long as distancing and quarantines remain in effect. In the Iometrics and Iometrics Global Workplace Analytics (2020), 88% of respondents were working from home regularly during the pandemic. This increase in telework due to COVID-19 has accelerated the trend toward more telework options (Kniffin et al., 2021).

Telework has been associated with some benefits such as flexible working, higher productivity, and autonomy (Gajendran and Harrison, 2007; Hill et al., 2010; Martin and MacDonnell, 2012). However, it has also been correlated with challenges such as social isolation and reduced job engagement (Sardeshmukh et al., 2012; Allen et al., 2015). The articles we reviewed confirmed these earlier results. Some employees benefitted from the positive side due to flexible working hours (Diab-Bahman and Al-Enzi, 2020; Sethi and Saini, 2020), but others reported more disruption or work-home conflict (Bolisani et al., 2020). These findings indicate that the existing drawbacks have not been overcome although discussion about continuing telework has persisted. Moreover, ineffectiveness of the traditional telework system in managing work and necessity for novel approach of telework was reported (Golden, 2009), but the pandemic brought more challenges for telework in work management. In particular, organizations that were faced with a sudden transition to telework during COVID-19 often lacked adequate preparation for a proper response to implement and manage telework, so managing employees’ work may have been even more inefficient.

Individuals also lacked the necessary transition time to prepare for telework. The studies in this review indicated that many employees experienced work-home conflict that was different than previous studies indicating that telework reduces conflict (Raghuram and Wiesenfeld, 2004). The difference is that the recent transition to telework was not voluntary for most employees. Employees who had no previous experience working from home experienced unexpected adverse effects, and accompanied uncertainty about working from home (De Klerk et al., 2021). The absence of natural boundaries introduced new difficulties in the distinction between their work and home life, so sustaining a work-life balance became a challenge (De Klerk et al., 2021). Therefore, to reduce work-home conflict, the sample articles have commonly discussed that individuals need to separate work in their home space, and organizations need to add support for employees’ adaptation to this new work environment.

### Perceptions of COVID-19

The Perception of COVID-19 theme mainly centers on how employees think and feel about COVID-19 issues (Table 3). Employees’ performance and productivity strongly rely on how they think and feel about their present work conditions and environment (Zareen et al., 2013). Consequently, perceptions of COVID-19 were generally described in the studies as a principal independent variable for various relationships with other constructs that were primarily related to employees’ work performance and commitment in their changing circumstances.

**TABLE 3:** Articles on perceptions of COVID-19.

Sub-themes	References	Research objective
Impact of perceived COVID-19 anxiety on work performance	Hu et al. (2020)	To advance a model explaining how the mortality salience caused by COVID-19 that influences behavioral and psychological responses
	Kang et al. (2021)	To examine how COVID-induced stress affects trust in the organization, job satisfaction, and commitment in tourism and hospitality fields
	Maity et al. (2020)	To quantify the influences of COVID-19 on private-sector employees in India
	Toscano and Zappalà (2020)	To establish a correlation among social isolation, stress, and remote work satisfaction
	Trougakos et al. (2020)	To build a model elucidating how COVID-19 anxiety impacts work, home, and health outcomes, and to analyze the role of problem-focused coping behavior to alleviate the harmful effects of COVID-19 anxiety
	De Clercq and Pereira (2021)	To examine behavioral responses to perceived COVID-19 threats by engaging in productive communication and debate with organizational colleagues
	De los Santos and Labrague (2021)	To analyze the fear of COVID-19 among nurses in community settings
	Liu et al. (2021)	To determine whether and when an employee’s perceived crisis strength affects work engagement and taking charge at work, and to assess the results of organizational interventions to mitigate the negative impact of COVID-19 on employees’ work
	Savolainen et al. (2021)	To explore psychological, contextual, and demographic predictors of COVID-19 anxiety

By classifying the common themes related to employees’ negative thoughts of the pandemic, this review identified how concerns about the present crisis influenced employees’ performance. In general, most employees reported negative feelings about the pandemic. Employees commonly reported a negative view of the pandemic and considered it a threat that produced fear (Maity et al., 2020; De Clercq and Pereira, 2021; De los Santos and Labrague, 2021), anxiety (Trougakos et al., 2020; Liu et al., 2021; Savolainen et al., 2021), or even mortality salience (Hu et al., 2020). Moreover, these negative psychological responses seemed to negatively affect employees’ job engagement and performance (Toscano and Zappalà, 2020; Kang et al., 2021).

COVID-19 anxiety tended to lead to a broader range of negative feelings for employees including fear and threats. Savolainen et al. (2021) designed a longitudinal survey to determine the characteristics of predictors that induced anxiety from COVID-19 for Finnish workers. The results showed that psychological factors, such as perceived loneliness, psychological distress, and technostress, were the most significant factors leading to employees’ perceptions of anxiety related to COVID-19 (Savolainen et al., 2021). Negative feelings and stress usually came from hardships in adapting to new work settings and social isolation. Similarly, Toscano and Zappalà (2020) revealed that social isolation raised employees’ stress and harmed their productivity in a remote work setting. These findings support previous studies showing that the lack of social interaction is a main reason for employees’ greater fear and stress (Weinert et al., 2015).

Employees with close contact with COVID-19 patients, called ‘frontline workers,’ include those in the medical sector as well as hospitality and tourism workers. Employees in these fields shared similar interests and challenges such as enduring the fear of directly interacting with patients and customers which increased their risk of contracting the disease (Kang et al., 2021). Liu et al. (2021) confirmed this negative correlation between the perceived impact of COVID-19 and frontline health workers’ work engagement. Frontline nurses working in a community setting also reported the strong influence of COVID-19 fear on their job satisfaction and turnover intention (De los Santos and Labrague, 2021). Given that frontline workers played a crucial role during the pandemic, workers in frontline settings have demanded that they be allowed to manage their negative outlook on the present situation.

Many scholars examined protective factors that could soothe the adverse impact of COVID-19 perceptions on employees’ job behavior and consciousness. Hu et al. (2020) and Liu et al. (2021) similarly elucidated that fear of mortality could be mitigated when individuals realized the meaning of a job and their usefulness as members of an organization. Trougakos et al. (2020) also identified the importance of both emotional fulfillment and proactive actions as relevant coping behaviors that could minimize threats and anxiety when confronting problems.

Although most articles described the negative consequences of COVID-19 anxiety, one article presented a different perspective on employees’ negative views (De Clercq and Pereira, 2021). The article claimed that negative emotions related to COVID-19 could foster employees’ creativity and lead to productive discussions. De Clercq and Pereira (2021) also stressed that perceived threats from the current circumstances could stimulate people to participate in positive responses to conflict in an emergency situation. Employees may be willing to expend energy to seek better, more innovative solutions by considering work-related conflicts as problems to overcome. Based on this argument, employees can accept the current situation in a positive way to recover from their reduced job engagement.

Several articles indicated that how employees perceive the *status quo* determines whether they can adapt to transformative work conditions and focus on their job performance, given that employees’ perception of crisis circumstances is an important factor affecting their job performance (Hu et al., 2020; Toscano and Zappalà, 2020; Trougakos et al., 2020; Liu et al., 2021). Several considerations are associated with social and emotional support to help employees overcome the negative influences of COVID-19 anxiety. Thus, the reviewed studies recommended that organizations manage employees’ perceptions to help them control their emotions and improve work efficiency.

Another finding revealed that how employees accepted and dealt with the pandemic strongly affected their work engagement during COVID-19 as much as changes in their work and life (Hu et al., 2020; Kang et al., 2021; Liu et al., 2021). Similar to the SARS and swine flu pandemics, a large number of healthcare workers experienced fear, anxiety, and frustration during the COVID-19 pandemic, so they had difficulties continuing their jobs (Hall et al., 2003; Ives et al., 2009; De los Santos and Labrague, 2021; Liu et al., 2021). However, workers’ job behaviors and attitudes can vary depending on whether they perceive that their current working environment is a personal threat. For example, Ives et al. (2009) reported that some workers believe that abandoning work would not always guarantee that they could protect themselves or their family’s safety in disastrous situations. In fact, most nurses had to regularly manage their negative emotions related to disease-induced anxiety and they found it difficult to work efficiently, which diminished their wellbeing (Hall et al., 2003). Studies on natural disasters like earthquakes have reported similar conclusions about the relationship between disaster-related perceptions and employees’ job engagement (Kim et al., 2020).

Despite the similarities, COVID-19 is different from previous disasters in its duration and global scale, resulting in a paradigm shift worldwide (Vaziri et al., 2020; Webb et al., 2020). The COVID-19 pandemic has raised the level of all employees’ general anxiety and severely reduced employees’ productivity (Liu et al., 2021). Thus, the reviewed studies strongly recommended that organizations must manage employees’ perceptions of COVID-19 to raise productivity. Organizations can help employees properly appraise the pandemic situation as a challenge and take appropriate coping strategies.

### Employee Wellbeing

Unlike the second theme focusing on immediate psychological reactions to COVID-19, the articles under this theme dealt with the overall consequences of COVID-19 for employees and organizations. Most of the reviewed articles on Employee wellbeing centered on employee adjustment, employee mental health, and social support (Table 4). According to Warr (1987, p. 24), job-related wellbeing is a holistic feeling based on two axes of pleasure and excitement about one’s job. Based on Warr’s words, Taj et al. (2020) noted that employee wellbeing entails a worker’s wellness of operations and feelings of belonging to an organization.

**TABLE 4:** Articles on employee wellbeing.

Sub-themes	References	Research objective
(1) Employees’ adjustment	Carnevale and Hatak (2020)	To discover the challenges and opportunities related to COVID-19 in HRM fields
(2) Employees’ mental health	Chersich et al. (2020)	To understand the infection risks and mental health challenges during the pandemic among healthcare workers
	Dinibutun (2020)	To estimate the prevalence of burnout among physicians and examine burnout factors
	Kuo et al. (2020)	To investigate work stress perceived by hospital staff and its influencing factors during COVID-19 in Taiwan
	Obrad (2020)	To identify the problems and interventions perceived by Romanian teachers during COVID-19
	Park et al. (2020)	To identify protective factors against psychological distress among infectious disease physicians coping with COVID-19
	Teng et al. (2020)	To confirm quarantine hotel employees’ depression, anxiety, and stress caused by COVID-19 in China
	Meseguer de Pedro et al. (2021)	To estimate the quarantine influence on self-perceptions of health and psychological capital, and to analyze the relationship with burnout
	Rodríguez-López et al. (2021)	To assess mental workload and burnout levels, and to identify increasing pandemic challenges for Spanish fashion retailers
3) Social support	Chong et al. (2020)	To understand how the pandemic influences employees and employers can alleviate the effects of COVID-19 task setbacks
	Bulińska-Stangrecka and Bagieńska (2021)	To identify the correlation between interpersonal relations, job satisfaction, and trust among Polish IT employees

Adjustment has a positive relationship with workers’ wellbeing (Vojnovic et al., 2014), whereas employees with adjustment problems appear to suffer. Carnevale and Hatak (2020) explored challenges and opportunities in terms of employees’ adjustment and wellbeing during COVID-19 and found that human resource management challenges during the pandemic stemmed from a mismatch between employees’ need for certain work conditions and their actual work environment. They recommended that organizations should understand the loneliness of single or childless employees and support them, so they do not feel excluded. Through these efforts, organizations can help employees adjust properly, and doing so can promote employees’ wellbeing.

Employees’ mental health is also related to wellbeing (Warr, 1987, p. 40–46). Although non-frontline workers were not forced to have close contact with infected patients, it turns out they also experienced mental health issues. Some studies reported employees’ worsening perceptions of their health, psychological capital, burnout, emotional exhaustion, and mental workload (Obrad, 2020; Meseguer de Pedro et al., 2021; Rodríguez-López et al., 2021). For Spanish fashion workers, there was a significant difference in the level of mental workload and burnout by job types or gender (Rodríguez-López et al., 2021).

Meseguer de Pedro et al. (2021) also surveyed Spanish workers and found that their perceived health decreased, and psychological capital increased while adhering to quarantine measures. Based on a survey by Obrad (2020), Romanian teachers perceived stress and constraints in an online teaching environment due to difficulties maintaining their health and lack of a digital learning infrastructure. These constraints increased the teachers’ stress levels and decreased work engagement and resilience. In contrast, Obrad (2020) also reported that work engagement increased support, which in turn increased resilience.

Some research on frontline workers discussed vulnerable work conditions. Frontline workers faced many challenges during the COVID-19 pandemic including direct contact with infected patients, the burden of patient care, and fighting against the lack of personal protective equipment and isolation rooms (Chersich et al., 2020; Kuo et al., 2020; Park et al., 2020). Especially in Africa with insufficient international support, countries lacked an adequate workforce and required low-cost sanitizers and water for handwashing (Chersich et al., 2020). As a result of research on psychological distress among South Korean physicians, Park et al.’s (2020) recommended more scientifically verified support and guidelines to properly cope with COVID-19.

Under these circumstances, healthcare workers and hotel employees in quarantine hotels working on the frontlines also exhibited mental and work stress, depression, post-traumatic stress disorder, anxiety, and burnout symptoms (Chersich et al., 2020; Dinibutun, 2020; Kuo et al., 2020; Park et al., 2020; Teng et al., 2020). These authors reported that these workers feared COVID-19 infection and transmission (Kuo et al., 2020), which made healthcare workers feel stressed about catching and spreading the virus to their loved ones (Chersich et al., 2020). Healthcare workers especially reported that they feared being ostracized by other people (Chersich et al., 2020). However, the researchers found that psychological challenges and mental states significantly depended on employees’ demographic characteristics such as age, income, having children, type of job, gender, and education level (Dinibutun, 2020; Kuo et al., 2020; Park et al., 2020; Teng et al., 2020).

Two articles examined how social support influenced employee wellbeing during the pandemic. Bulińska-Stangrecka and Bagieńska (2021) studied the positive effect of relationships among employees on job satisfaction in the Polish IT industry. In addition, this effect was mediated by interpersonal trust among team colleagues and with managers. They noted that interpersonal trust is a social bond between colleagues and managers, and job satisfaction is part of employee wellbeing and positive mental health (Rose et al., 2017; Bulińska-Stangrecka and Bagieńska, 2021). Chong et al. (2020) indicated that the sense of connectedness between individual workers alleviated exhaustion levels and helped employees’ wellbeing. They also found that the level of perceived organizational task support for telework as a social cue moderated the relationship between current exhaustion and future withdrawal.

In light of the mostly pessimistic outlook of COVID-19, the pandemic has exacerbated the employees’ adjustment, job satisfaction, and mental health challenges (Islam et al., 2020). Considering that the dynamic interplay between individuals and their environment affects psychological wellbeing in the long-term (Folkman, 2008), it is understandable that employee wellbeing was ultimately pertinent to employees’ perceptions of COVID-19. Thus, to enhance employee wellbeing, organizations need to consider how their employees perceive COVID-19. Additionally, given that social support is a vital element for employee wellbeing, social support is needed to increase job satisfaction and mitigate mental problem symptoms.

The reviewed studies indicated that most employees, regardless of the sector in which they worked, experienced similar situations where they had to endure negative emotions due to social distancing policies during the COVID-19 pandemic. In the recently published studies, frontline workers’ experiences were reported more frequently as they commonly experienced psychological distress, emotional anxiety, and acute depression (Campbell and Gheorghe, 2021; Mitchell and Lăzăroiu, 2021; Pera and Raluca-Ştefania, 2021) although these articles were not included in our original search. Creating a receptive and cooperative organizational culture can encourage employees to decrease the negative effects of the pandemic on employee wellbeing (Di Fabio and Kenny, 2019; De Clercq and Pereira, 2021). Thus, organizations need to deal with individual employees’ wellbeing and manage the whole organizational climate during the pandemic.

To improve both individual and organizational wellbeing, previous scholars have recommended that organizations encourage employees to share their own positive psychological capital with their colleagues (Luthans et al., 2008). Positive psychological capital such as resilience or hope has also been found to be related to employee wellbeing and positive organizational behavior (Avey et al., 2010) that can contribute to building a favorable organizational climate. Numerous studies have recommended that organizations should consider an emotional and relational approach for employees to encourage employee’s beneficial psychological capital (Di Fabio and Kenny, 2019). Social support is also beneficial for organizations as they work to foster a supportive organizational culture where employees can enhance their own positive psychological capital and share it with others (Nahum-Shani et al., 2011). Thus, creating an organizational culture with reciprocal social support among employees can raise both employee wellbeing and organizational productivity in this harsh era.

### Organizational Strategies

Ten studies examined specific strategies at the organizational level and determined whether these strategies remained valid during the COVID-19 period (Table 5). One study also focused on how employees’ perceptions of the strategies affected their organizational behaviors. The sub-themes included leadership strategies, organizational human resources strategies, organizational management strategies, and employees’ perceptions of organizational strategies.

**TABLE 5:** Articles on organizational strategies.

Sub-themes	References	Research objective
(1) Leadership strategies	Bekirogullari and Thambusamy (2020)	To investigate how small businesses maintained their operations through virtual leadership during COVID-19
	Dirani et al. (2020)	To explore the roles of leaders and organizations, and to elucidate the novel role of HRD during the COVID-19
(2) Organizational HR strategies	Gan (2020)	To recommend scholarship regarding baby boomer nurses who delayed retirement to help bedside nursing
	Jankelová et al. (2021)	To explore the impact of crisis management competencies in healthcare facilities to manage the crisis
	Tanpipat et al. (2021)	To assess the influence of organizational norms on remote work productivity and commitment
(3) Organizational management strategies	Akingbola (2020)	To examine the impacts of COVID-19 on non-profit employees and HR management
	Bieńkowska et al. (2020)	To confirm the organizational reliability model and explore its operation
	Buhusayen et al. (2020)	To explore frontline managers’ challenges of operations’ turnaround management strategies to deal with COIVD-19
	Dwomoh et al. (2020)	To assess hoteliers’ human resource strategies for maintaining their businesses during the COVID-19 in Ghana
	Sharma (2020)	To explore organizational challenges of enterprises, labor, and employees
(4) Employees’ perceptions of organizational strategies	Manuti et al. (2020)	To explore employees’ perceptions of sustainable HRM in the COVID-19, and the extent to which employees’ views on organizational support could affect beneficial organizational behaviors

Two articles discussed leadership as an essential requirement to better cope with the pandemic. Dirani et al. (2020) recommended that leaders should emotionally and interpersonally support employees experiencing trauma, and also promote emotional stability and employees’ wellbeing. Leaders should also innovate communication systems that best meet the new work conditions during COVID-19 as well as endeavor to maintain the organization’s financial health (Dirani et al., 2020). Virtual leadership for small businesses was also discussed. Bekirogullari and Thambusamy’s (2020) integrative literature review identified challenges such as using unfamiliar digital technologies, having difficulty with virtual communication, and employees being distracted during work. They also proposed alternatives to overcome these constraints, such as choosing proper communication strategies and creating elaborate communication protocols. Leaders were urged to be more accommodative to employees’ preferred ways of communication to address their struggles and help them adapt to the virtual environments (Bekirogullari and Thambusamy, 2020).

Strategies for managing human resources are critical for corporations to improve overall organizational performance and to promote further growth and help employees be engaged for both individual and organizational progress. The studies confirmed that an important aspect of organizational strategies is providing sufficient guidelines and encouraging communication. Tanpipat et al. (2021) surveyed Thai employees in various corporate environments and found that organizational norms surrounding remote work played a significant role in helping employees increase productivity and organizational commitment. Gan (2020) found that nursing personnel benefitted from telework during the pandemic because telework made it possible for them to work during flexible hours. The extension of baby boomer nurses’ work (i.e., past retirement age) was also advantageous for both younger bedtime nurses with less experience and hospitals to compensate for the shortage of nurses during the pandemic (Gan, 2020). In terms of adopting appropriate strategies, Jankelová et al. (2021) recommended that managers need proper crisis management abilities, including effective decision-making and cooperative communication skills that encourage teamwork. In particular, they confirmed the mediating effect of sharing information, teamwork, and cognitive diversity, and asserted that abundant information and directions helped employees feel safer and raised their productivity during the pandemic.

Five studies examined the need for organizational strategies for maintenance and management. Bieńkowska et al. (2020) suggested that in a crisis, maintaining the previous work tasks and how the organizations ran the business reduced overall organizational reliability. Thus, they suggested that managers should redefine employees’ tasks and job procedures and allow the organization to adapt to the environmental changes. Buhusayen et al. (2020) also emphasized that frontline managers should be involved in the management planning stage and share information about implementing turnaround management strategies. Akingbola (2020) explored the impact of COVID-19 on non-profit employees and the need for management strategies such as scenario planning that reflects employees’ voices and challenges. However, layoffs, as an organizational strategy during COVID-19, limited their human resources strategy choices when there was no government support to keep them afloat (Dwomoh et al., 2020). Particularly small companies were not prepared to manage work when they faced severe COVID-related situations and struggles (Sharma, 2020).

One study focused on how employees recognized the strategies that were adopted by their organizations. Based on a survey by Manuti et al. (2020), if employees perceived that human resource management involvement worked well, their organizational engagement, extra-role behavior, and ability to cope with organizational change were likely to be better. In particular, coping with organizational change partially mediated the perception of the influence of human resource management commitment on organizational engagement and extra-role behavior.

Organizations also implemented various strategies related to leadership, human resources, and management plans to survive during COVID-19 and in the post COVID-19 era. These strategies must be formulated and properly applied with comprehensive perspectives on diverse dimensions. Thus, most studies encouraged organizations help their employees and managers to work more productively, and support the creation of an amicable team atmosphere. If an organization is not viable, it is difficult for the organization to execute survival strategies. In addition, informing employees about helpful company policies is important as this procedure can encourage employees to engage in more positive organizational behaviors.

Organizations have used various strategies to respond to a crisis including managerial leadership, management strategies, and employee development practices. Our results showed that employees needed support in many ways, and leaders needed to help them stay emotionally stable and boost their wellbeing using reasonable strategies to care for them. Bojadjiev and Vaneva (2021) noted that the roles leaders played in the crisis were important for effective organization operation, good communication, and collaboration. Especially in a virtual environment, hierarchical leadership negatively affects team performance (Hoch and Kozlowski, 2014). Leaders should understand and empathize with their team members rather than maintaining traditional leadership.

The results of the reviewed studies indicated that organizations need to pay attention to how to implement approaches in reaction to COVID-19. In particular, organized norms at the organizational level tend to play an important role and sufficient guidelines were needed (Tanpipat et al., 2021). For example, organizations needed to provide telework guidelines on what organizations would do and what workers should anticipate from work (International Labour Organization, 2020). In addition, Van Zoonen et al. (2021) found that specific criteria allowed employees to objectively track their progress and adjust their performance in a telework environment where employees lacked immediate feedback. However, unclear evaluation criteria induced uncertainty about performance expectations (Van Zoonen et al., 2021). They recommended that organizational norms and guidelines should be provided to reduce uncertainty during the pandemic.

The results also revealed that sharing information about organizational strategies can help employees feel safer in crises. Organizational support delivers messages of assurance for employees that their wellbeing is important (De Klerk et al., 2021). Such messages also increase the level of job resources to reduce employees’ job strain, based on the job demand-resource model (Bakker and Demerouti, 2007; Khodakarami and Dirani, 2020). Congruence of values between organizations and employees can also lead to the same goals even in virtual environments (Gelle-Jimenez and Aguiling, 2020). Given that shared values positively affect organizational performance (Jung and Avolio, 2000), it is important to not only establish strategies that are appropriate for the situation, but to also share the strategies with employees.

### Influences on Career Behaviors

Employees have faced drastic changes in their work environments and lifestyles that have caused unprecedented difficulties that threaten their sense of staying safe and improving their job performance. Consequently, many employees have changed their career behaviors in response to the pandemic. Three articles discussed the influences on career behaviors during the pandemic and they can be divided into two groups: the decision to be absent and career decisions (Table 6).

**TABLE 6:** Articles on influences on career behaviors.

Sub-themes	References	Research objective
(1) The decision to be absent	Gregori (2020)	To examine absenteeism in companies in Romania during the COVID-19 pandemic
(2) Career decisions	Dos Santos (2020)	To elucidate the nursing shortage and how COVID-19 has affected nursing students’ decisions in South Korea
	Hoang (2020)	To determine the effect of expat teachers’ attitudes about local policies, and how their social engagement impacted their intention to leave their working country due to COVID-19

Absenteeism is an important problem for all organizations in that absent employees generally affect many aspects of individual and organizational performance. Employee absenteeism occurs when an employee fails to be present during regular work hours (Duclay et al., 2015). Gregori (2020) found that factors influencing employees’ absenteeism morphed into more attitudinal and personal issues during COVID-19. More specifically, satisfaction with an organization’s safety and employee wellbeing measures, financial benefits, and work-life balance reduced employees’ intention to be absent (Gregori, 2020). When workers needed to keep working despite the threat of infection, organizational endeavors to make the organization a safer, better working environment were a key motivation for employees not to leave their jobs.

Employees’ decisions are not limited to their present working intentions. Further, pre-workers and workers can decide to change workplaces or quit in preparation for a second career for various reasons related to safety and wellbeing. Dos Santos (2020) stressed that financial consideration was the most important motivation for nursing students to choose medical jobs, but the low salary compared to the risk of infection was one reason many nursing students decided not to become nurses. However, Hoang (2020) argued that expatriate teachers’ motivation to leave their host countries directly depended on both the countries’ safety measures for foreign workers and the social support of the local community.

It is necessary to understand how workers and pre-workers responded to the pandemic because it is closely related to employee retention. Employees tend to modify their career behaviors considering their current working conditions and organizational support. A study revealed that employees chose to be absent when they thought their organization had not tried to create a safe work environment (Gregori, 2020). In addition, they may have attempted to change their job conditions based on the national and organizational support, which could allow them to safely continue their work to earn a living. Employees’ career behavior is determined by the extent to which organizations guaranteed work conditions and care for employee wellbeing. Thus, changes in employees’ career behaviors are based on how they perceive their own wellbeing and organizational strategies.

The findings from this literature review indicated that work setting, evaluation of employee’s general work and life, employee wellbeing, and organizational strategies can be significant factors that shape employees’ work attitudes and behaviors. Previous research has confirmed that if employees are not satisfied with any of the elements, their organizational commitment level will be lower and they are more likely to withdraw from work through absenteeism or turnover (Podsakoff et al., 2007).

Given that crisis conditions are not controllable for individuals without organizational management guidance, employees’ job attitudes depend on their assessment of organizational support for employee’s safety and wellbeing (Mosadeghrad, 2013; Russo et al., 2013). Since an employee’s decision to be absent or decide to quit has a negative impact on organizations for employee retention and profit (Ongori, 2007), organizations need to be aware of why employees change their career behaviors and carefully address the underlying issues, especially during COVID-19.

**FIGURE 2:**
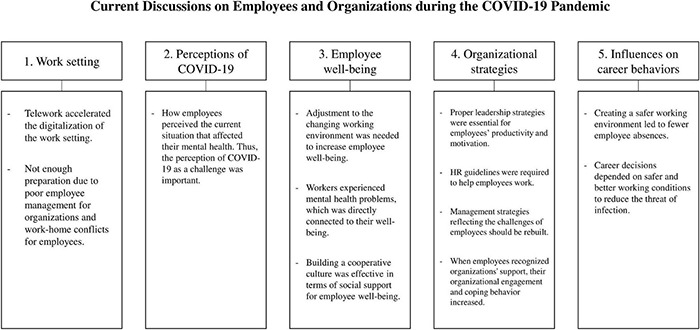
Main findings.

Employees’ motivations for absenteeism and turnover are pertinent to a match between personnel needs and organizational demands (Hongvichit, 2015). Organizations need to encourage employees to think about their better future career expectations as well as believe in their ability to persist at work despite risky conditions (Brown and Lent, 2017). These attitudes can prevent employees from the intention to be absent or quit. Therefore, the sample papers have encouraged organizations to endeavor to understand employee’s personal needs and take action to match the employees’ needs and those of the organization. Figure 2 summarizes the findings of our study.

## Implications

### Theoretical Implications

The theories used to explain employees’ and organizations’ circumstances during COVID-19 were not frequently discussed in the reviewed literature. However, a few theories under each theme were presented to develop scholarly arguments on such issues.

Event system theory was useful to address work settings during COVID-19. It explains that the intensity of an event reflects the novelty, disruption, and criticality, and the more these characteristics are present, the more likely they will affect behaviors (Morgeson et al., 2015). Thus, changing the work environment could affect employees’ perceptions of telework and employees’ behaviors differently depending on how novel, disruptive, and critical employees perceive their own context. When applying this theory to changes in new work settings due to COVID-19, the degree of novelty is different depending on the previous experience of working from home. For example, employees in knowledge-intensive industries were more likely to use telework before the pandemic compared to workers in other fields (Abulibdeh, 2020). In addition, there was a difference in workers’ level of disruption depending on whether they could continue their work normally. Some employees did not experience much disruption, but rather were more productive due to flexible working hours (Diab-Bahman and Al-Enzi, 2020; Sethi and Saini, 2020). However, Bolisani et al. (2020) reported that the respondents did not always think it was positive because they were unprepared for the distracting environment and autonomy working from home and lacked physical equipment. Lastly, the criticality of telework reflected how employees perceived telework as having a long-term impact. Some employees might believe that it is best to continue telework because the pandemic is continuing and the importance of telework will expand after the COVID-19 pandemic (Abulibdeh, 2020). Consequently, even if they face a similar event, employees will have different levels of challenges and event strength and different perspectives on how they will accept a new or ongoing crisis.

Employees’ awareness of the pandemic and coping strategies was explained by transactional stress theory (Trougakos et al., 2020; Liu et al., 2021). Emotions are systematically produced by transactions between the individual and the environment, which are mediated by the individual’s cognitive appraisal and coping process (Folkman, 2008). Cognitive appraisal relates to an evaluation process of circumstances and the environment of individuals based on their wellbeing. This assessment determines how individuals cope with and feel about demands placed on them. According to this transactional theoretical framework, employees evaluated the current crisis as a threat, and they had negative perceptions of COVID-19 such as anxiety and fear because the COVID-19 crisis brought sudden unique changes in their working environment that became stressful demands for employees. Coping represents adaptational outcomes when a person forms a perception of an event (Lazarus and Folkman, 1987). Coping strategies can vary based on how individuals view stressful circumstances as harm, threat or challenge (Goh et al., 2010). Most of the articles under the Perceptions of COVID-19 category indicated that COVID-induced negative emotions lowered employees’ engagement. However, one study (De Clercq and Pereira, 2021) argued that these pessimistic responses can sometimes encourage employees to improve their productivity and creativity. Once employees appraise the current crisis as a helpful challenge for themselves, they are willing to adopt coping actions that enhance their engagement.

Hobfoll’s conservation of resources (COR) theory was particularly used to support employee wellbeing during the COVID-19 pandemic (Chong et al., 2020; Meseguer de Pedro et al., 2021). The theory explains that people tend to protect and build resources perceived as valuable including objects and energy (Hobfoll et al., 2018), and the amount of retained resources is important (Chong et al., 2020). People consider change and transition to be stressful in that they can cause fluctuations in resources (Hobfoll et al., 2018). During the COVID-19 pandemic, everything abruptly changed from people’s lifestyles to work settings, and even employees’ safety was not guaranteed. Therefore, loss of resources for adaptation and stability was unavoidable. Working during the pandemic placed enormous demands on employees by continuously depleting resources and reducing employee wellbeing. People who lack resources are far more vulnerable to the continuous loss of resources when they work in stressful jobs for a long time or they do not have enough time to replenish surplus resources (Halbesleben et al., 2014). Especially, frontline workers should spend much more energy, and even draw their sense of vocational duty. As a result, they experienced resource deterioration (Meseguer de Pedro et al., 2021), felt despair and helplessness, which led to self-defeating consequences such as burnout (Dinibutun, 2020). Moreover, when organizations do not provide enough resources, employees cannot enhance their work productivity (Vaziri et al., 2020). A mismatch between employees’ needs and organizations’ support can also contribute to severely lower employee wellbeing (Mostafa, 2016). Social support can also be explained by resource investment and recovery mechanisms (Halbesleben et al., 2014). Spending time and energy to socially support colleagues means a loss of resources for employees in the short-term, but this investment can be returned in various forms from colleagues in the long-term. Thus, in a crisis when employees cannot avoid resource deterioration, strengthening interdependence and increasing communication to share difficulties with their co-workers can help protect employees’ wellbeing.

The job demands-resources (JD-R) theory explains why organizational strategies as a job resource support employees suffering from job demands (Bakker and Demerouti, 2007). Job resources such as the organization at large or social relations can lead to high organizational outcomes by acting as the motivation and can buffer the effect of job demands on strain such as job-related anxiety or health complaints (Bakker and Demerouti, 2007). Two articles in our review on leadership strategies argued that leaders should support employees both emotionally and interpersonally (Bekirogullari and Thambusamy, 2020; Dirani et al., 2020). In line with the JD-R model, leaders can positively influence employees by offering social support (Bakker and Demerouti, 2017), and communication with colleagues is especially critical. In addition, organizational norms and adequate guidelines help employees by increasing role clarity and task identity, which can be job resources. For similar reasons, sufficient information and participation in decision making can also help employees be more productive. In contrast, isolation (Toscano and Zappalà, 2020), technostress (Bekirogullari and Thambusamy, 2020) and organizational changes (Manuti et al., 2020) were aspects of job demands during the pandemic and consequently led to low work engagement or low productivity. Therefore, it is important that employees clearly understand their organizations’ strategies. In particular, human resources involvement worked as a job resource during the pandemic (Manuti et al., 2020), and has been shown to reduce the impact of job demands on strain (Bakker and Demerouti, 2007). Thus, organizations should try to reduce the job demands and determine how to encourage their employees.

Lent et al. (1994) suggested that career-related choices and actions are created through the dynamic interplay between one’s self-efficacy, job interests, and outcome expectations based on social cognitive career theory. Two studies reported that employees decided to change their career behaviors (e.g., to be absent, to leave the countries where they were working) because they anticipated negative outcomes if they continued working in the current conditions (Gregori, 2020; Hoang, 2020). Without safety measures and social support from governments and organizations, employees cannot engage well in their job tasks and thus may decide to change their job. Dos Santos (2020) found that most nursing students considered financial concerns as the most important reason for leaving their career paths. According to Brown and Lent (2017), self-efficacy and outcome expectations affect career-related actions far more when career interests are deeply interrelated with economic motivations. Consequently, the choice to keep studying for a nursing career intensely relied on whether students perceived that they could earn much money by enduring the vulnerable working environment or not. Self-efficacy and outcome expectations are an individual’s cognitive process, which interacts with his or her surrounding environment. The articles related to self-efficacy were all relevant to how employees perceived their surrounding circumstances including financial rewards (Dos Santos, 2020), organizational or governmental support (Gregori, 2020; Hoang, 2020), and employees’ own safety (Gregori, 2020). They also discussed how employees could adapt in the changing pandemic setting by reorganizing their career choices and goals. These findings illustrate that employees consider not only their future career expectations, but also whether they can endure and persist to work in risky situations.

Considering that changes induced by COVID-19 are complicated and difficult to describe with one simple theory, we can elucidate that employees and organizations were susceptible to the COVID-19 context and complex changes caused by the pandemic. Therefore, future studies on employees and organizations in crises such as during the COVID-19 pandemic should be conducted with a holistic perspective examining multiple relationships for each phenomenon and revealing results. By understanding this holistic organizational perspective, research can contribute to a deeper understanding of the complexity of COVID-induced problems and counteract multiple difficulties for employees and organizations. By building a crisis prediction process with a holistic theoretical foundation, employees and organizations can better confront future crises.

### Practical Implications

From a practical perspective, both employee- and organizational-level practices have been needed to judiciously adapt to the COVID-19 crisis. At the employee level, considering new forms of work are emerging due to technological developments and social changes, employees should endeavor to build and maintain capabilities to create a productive environment where they can work. Employees should also create their own independent spaces by establishing physical boundaries between their home and workspaces. In terms of employees’ behavioral readiness, employees need to engage in appropriate crisis-coping behaviors since it helps employees believe that they can have some level of control over the circumstances with effective, problem-focused actions (Trougakos et al., 2020). In terms of psychological readiness, resilience and optimism can serve as protective factors to help employees withstand difficult circumstances. Creating an environment with low conflict and high enrichment in the workplace can help overcome these challenges. It would also be helpful to pay attention to the relationships with managers and colleagues by sharing emotions and encouraging each other. Therefore, adequate interactions with others should be learned and encouraged. Further, from individual efforts to readiness, employees need to know that organizations are making efforts to provide strategies to help them and utilize their external support.

At the organizational level, human resources should focus on creating better work conditions and provide proper guidance in unconventional work settings. Examples include work rearrangements, financial aid to purchase digital devices, and telecommunication systems. Moreover, organizations should establish strategies that reflect employees’ psychological states and challenges and help employees resolve the problems they face. Organizational concerns can positively influence employees’ mental health, and individuals are less likely to experience emotional difficulty when they think they will receive enough support from organizations. Therefore, various programs can be designed based on the difficulties associated with employee wellbeing in crisis situations, including support for mental health. In addition, organizations must communicate about their strategies to support employees. Last, additional organizational efforts to create an effective organizational culture with social support among employees should be highlighted. A leader’s capability to respond to a crisis, provide social support, and display servant leadership can help employees perceive that employers care about them, and this perception can relieve their stress level. Therefore, the roles of managers and organizational strategies in providing social support to create a positive organizational climate and wellbeing are important to address both employees’ wellbeing and organizational productivity in this crisis.

## Conclusion

This literature review of 45 recent studies confirmed that employees and organizations faced numerous challenges and incorporated diverse strategies to overcome the challenges during the crisis. COVID-19 is a new and different challenge causing suffering for companies and employees around the world. The organizations have incorporated environmental changes and have begun to develop workable strategies. These changes have meant transitioning to a new norm, but the findings also highlight the importance of employees’ psychological capital, making them resilient and helping them manage difficult situations. Thus, there is a need for organizations to build multi-dimensional strategies and a favorable organizational culture to address employees’ psychological responses to crises. In addition, to ensure successful execution of countermeasures, employees and organizations must be prepared for further changes.

Despite the meaningful results, this literature review has some limitations. Given the recent nature of COVID-19, the current review included a limited number of studies. Thus, there is still room to uncover additional phenomena by including the newest literature published after the completion of our literature search. Additionally, future studies can be tuned to deeply uncover the various impacts of the COVID-19 pandemic on diverse social groups by gender, race, and age. Some scholars have already reported interesting findings. Duncan (2020), Kirkman (2020), and Stevens (2020) have provided empirical evidence of gender differences in behavioral and psychological difficulties during the pandemic. Considering the importance of this topic from a perspective of inclusive management, more in-depth investigation is needed through both quantitative and qualitative data. Finally, to determine the long-term impact of the COVID-19 pandemic on individuals and organizations, longitudinal studies should be conducted.

## Data Availability Statement

The original contributions presented in the study are included in the article/supplementary material, further inquiries can be directed to the corresponding author/s.

## Author Contributions

All authors listed have made a substantial, direct, and intellectual contribution to the work, and approved it for publication.

## Conflict of Interest

The authors declare that the research was conducted in the absence of any commercial or financial relationships that could be construed as a potential conflict of interest.

## Publisher’s Note

All claims expressed in this article are solely those of the authors and do not necessarily represent those of their affiliated organizations, or those of the publisher, the editors and the reviewers. Any product that may be evaluated in this article, or claim that may be made by its manufacturer, is not guaranteed or endorsed by the publisher.
